# Health-related quality of life following cranioplasty: a cross-sectional cohort study – Cranio-PRO

**DOI:** 10.1007/s10143-024-03158-6

**Published:** 2025-01-14

**Authors:** Mohammad A. Mustafa, Christopher P. Millward, Conor S. Gillespie, George E. Richardson, Abigail L. Clynch, Sumirat M. Keshwara, John Doherty, Thomas Humphries, Abdurrahman I. Islim, Christian Duncan, Catherine J. McMahon, Andrew R. Brodbelt, Michael D. Jenkinson, Ajay Sinha

**Affiliations:** 1 University of Liverpool, Liverpool, UK; 2 The Walton Centre NHS Foundation Trust, Liverpool, UK; 3 Alder Hey Children’s NHS Foundation Trust, Liverpool, United Kingdom

**Keywords:** Cranioplasty, Quality of life, Patient reported outcomes, Qualitative

## Abstract

Cranioplasty is an operation that aims to repair a defect in the skull. Indications commonly include Traumatic Brain Injury (TBI), tumours, and infections. It carries a high rate of postoperative morbidity. Due to underlying pathology and the nature of the operation patients’ Health-related Quality of life (QoL) can be severely affected. Patient reported outcomes (PROs) are poorly understood following cranioplasty. To investigate PROs following cranioplasty a cross-sectional cohort study of adults undergoing cranioplasty between 01/03/2010–30/08/2020 was conducted. If consent was provided, participants were sent five health reporting questionnaires: a study specific questionnaire, EuroQoL – 5D – 5L (EQ-5D-5L), RAND Short-Form 36 (SF-36), Hospital Anxiety and Depression Scale (HADS), and Derriford Appearance Scale (DAS-24). 239 eligible patients were contacted and 72 (30.8%) participated. Median [Range] age was 52.5 [23 – 95] of which 59.7% (*n*= 43) were male. 16.9% (*n* = 12) were categorised with borderline and 29.0% *(n* = 20) with extreme depression, 15.9% (*n* = 11) reported borderline and 35.2% (*n* = 25) extreme anxiety. 69.1% (*n* = 47) had problems with usual activities and 71.6% (*n* = 49) suffered from pain/discomfort. Median (Range) EQ-5D-5L visual analogue scale score for overall health was 70.0 (5–100). 76.0% (*n* = 53) reported feeling conscious or having negative feelings towards their appearance. When compared to normative UK population, physical functioning, overall health, and energy/fatigue were the most affected. After cranioplasty, patients may have reduced HRQoL. Signposting and referral to well-being services could benefit patients’ care. Adequately powered longitudinal studies are required to further elucidate change in PROs following cranioplasty.

**Supplementary information:**

The online version contains supplementary material available at 10.1007/s10143-024-03158-6.

## Introduction

Cranioplasty is a neurosurgical procedure that aims to repair a physical defect in the cranium. The most common indications for a cranioplasty procedure include traumatic brain injury, infected bone flap from previous intracranial surgery, and a brain tumour which exhibits bone invasion [1]. A cranioplasty can be performed using a patient’s own bone (autograft) or using an artificial material (allograft). There is no general consensus on the optimum cranioplasty material, however allograft cranioplasty has been demonstrated to lead to better clinical outcomes [2]. Artificial cranioplasty materials include titanium, hydroxyapatite, PEEK (Polyetheretherketone)/PEKK (Polyetherketoneketone), PMMA (Polymethylmethacrylate) and porous polyethylene [3].

Cranioplasty carries a high risk of postoperative morbidity, with the rates of complications ranging from 8 to 30% [1, 4, 5]. Common post-operative complications include surgical site infection, wound breakdown, pain, and headaches. Morbidity from the operation often warrants a corrective procedure, leading to removal of the cranioplasty and subsequent insertion of a new one [6]. This can often lead to a patient undergoing multiple operations in addition to coping with their underlying pathology, exacerbated by a high risk of postoperative morbidity.

Patient-reported outcomes (PROs) are outcomes directly reported by the patients which help physicians measure the impact of a health condition and its treatment. Health-related quality of life (HRQoL) is a PRO that assesses how a patient’s physical, social, and emotional well-being are affected by a medical condition or treatment. HRQoL following cranioplasty is poorly understood, the primary reason for which is the lack of use of validated patient-reported outcome measures (PROMs) [7]. The primary objective of this study was to evaluate HRQoL following cranioplasty.

## Methods

### Study design

This was a single centre, cross-sectional observational study carried out at The Walton Centre National Health Service (NHS) Foundation Trust, Liverpool, United Kingdom. The study was conducted according to the guidelines of the Declaration of Helsinki and approved by the Research Ethics Committee of the NHS Health Research Authority (IRAS: 291914).

### Participants

All adult participants (> 16 years) that underwent a cranioplasty between 1st March 2010 and 30th August 2020 with the ability to communicate effectively in English were eligible for inclusion. Participants were included for all craniectomy indications and cranioplasty materials. Participants were excluded if they did not have a current UK address and if they did not have the capacity to consent to participate in the study. Eligible participants were identified using a pre-existing database containing details of patients that underwent a cranioplasty operation. Patients’ addresses were obtained from the hospital electronic notes system, and prior to sending out study invitations, they were cross-checked using the NHS Spine service, which is directly linked to the patient’s primary care records.

### Consent process and questionnaire distribution

Eligible participants were contacted through post. All participants were first mailed a cover letter, participation information sheet (PIS), and a consent form. To confirm enrolment in the study, participants were asked to return a signed consent form using a study specific pre-paid return envelope. Participants were given four weeks to respond to the initial study invitation, and if no response was received, they were contacted a second time, through a repeat invitation consisting of the same cover letter, PIS, and consent. Finally, if no response was received to either consent form, participants were invited to participate via telephone. Participants that returned completed consent forms were then sent a set of five questionnaires. All returned consent forms and questionnaires were filed in a secure hospital cabinet.

### HRQoL questionnaires

The RAND 36-Item Short Form Health Survey (SF-36) 1.0 [8] was chosen as it is a widely used, generic HRQoL assessment tool. It contains 36 items split into eight HRQoL domains. Total questionnaire scores range from 0–100, with a higher domain score indicating a better health state.

The Euro-QoL-5D-5L (EQ-5D-5L) [9] is a tool that has been used to assess HRQoL in clinical trials, population studies, and real-world settings. It consists of two sections: The EQ-5D descriptive system which assess five HRQoL domains and the EQ visual analogue scale (VAS) which is a 100-point scale with a higher value indicating better overall health.

The Hospital Anxiety and Depression Scale (HADS) [10] was chosen as it is a standardised 14-point questionnaire which has been previously used to detect mild to severe levels of anxiety and depression. A higher score in each domain indicates a higher level of anxiety/depression.

The Derriford Appearance Scale (DAS) [11] was chosen as it is a tool that helps investigate contributing factors to appearance problems. It is known to have a high level of reliability and validity and has previously been used in clinical (plastic surgery, oncology, and psychology) settings. A higher overall score indicates a more negative feeling towards one’s appearance.

### Clinical details

Clinical details for all included patients were available in a pre-existing dataset forming an institutional cranioplasty case series. Clinical characteristics were collected and recorded at the time of the cranioplasty. Patient sex, age, craniectomy indication, WHO performance status, Age-Adjusted Charlson Comorbidity Index (ACCI), cranioplasty material and postoperative status were collected.

### Statistical analysis

Statistical analysis was completed using IBM SPSS Statistics (Version 26.0, Released 2019, IBM Corp., Armonk, NY) and R studio Version 1.4.1103 (RStudio). Descriptive statistics were performed to summarise variables, these were expressed as frequencies and percentages. Continuous data was subject to a Shapiro–Wilk test of normality, with normally distributed data being presented as mean with standard deviation (SD) and skewed data being presented as median with an inter-quartile range (IQR). Depending on the distribution of data, continuous variables were compared using a student’s t-test or a Mann–Whitney U test, and categorical variables using a Chi-Squared Test. A p-value < 0.05 was considered statistically significant. One-way ANOVA test and independent sample Kruskal–Wallis Test were performed for parametric and non-parametric data, respectively, to assess the relationship between a single factor and variable. Multiple imputation was not performed to account for missing data.

## Results

### Study invitation and questionnaire distribution

The pre-existing cranioplasty dataset contained 279 patients. Forty patients were excluded as they were deceased, and five excluded as they did not have an available address. Therefore, 239 (85.6%) eligible participants were contacted. All participants were first contacted on the 8th of April 2021. A total of 104 (44.4%) participants consented to taking part in the study with 72 (30.8%) participants returning completed questionnaires.

### Patient population

The median age of participants was 52.5 years [Range: 23.0 – 95.0]. Most participants were male (*n* = 43, 59.7%). The most common indications for the initial craniectomy operation included traumatic brain injury (*n* = 25, 34.7%), tumour (*n* = 16, 22.2%), and infected bone flap (*n* = 11, 15.3%). The most common cranioplasty materials used were titanium plate (*n* = 19, 26.4%), hydroxyapatite (*n* = 17, 23.6%), and titanium mesh (*n* = 15, 20.8%). At the time of questionnaire completion, most participants were unable to work due to their health status (*n* = 20, 27.8%). Five (6.9%) participants underwent further surgery for cranioplasty removal. Patient demographic data is presented in Table 1.

**Table 1:** Demographics, baseline characteristics, and operative details for responders and non-responders

Variable	Sub-variable	Total number (%)		*P-value* (95% CI)
		Responders	Non-responders	
Number of patients		72	167	
Median age in years (Median) [Range]		51.9 [23.0–95.0]	47.5 [20.0 – 83.0]	0.033
Sex	Male	43 (59.7)	108 (64.7)	0.467
	Female	29 (40.3)	59 (35.3)	
ACCI (Median) [Range]		0.0 [0–5]	0.0 [0–6]	0.107
Indication for Craniectomy	Traumatic brain injury	25 (34.7)	61 (36.5)	0.434
	Tumour (any pathology)	16 (22.2)	32 (19.2)	
	Infected bone flap	11 (15.3)	30 (18.0)	
	Cerebral infarct	2 (2.8)	14 (8.4)	
	Intracerebral haemorrhage	3 (4.2)	9 (5.4)	
	Subarachnoid haemorrhage	3 (4.2)	6 (3.6)	
	Cystic lesion	3 (4.2)	6 (3.6)	
	Primary intracranial infection	1 (1.4)	3 (1.8)	
	Other	8 (11.1)	6 (3.6)	
Cranioplasty material	Titanium plate	19 (26.4)	40 (23.9)	0.773
	Titanium mesh	15 (20.8)	28 (16.7)	
	Acrylic	13 (18.1)	37 (22.2)	
	Hydroxyapatite	17 (23.6)	32 (19.2)	
	PEEK/PEKK	3 (4.2)	12 (7.2)	
	PMMA	5 (6.9)	16 (9.5)	
	Autologous	−	2 (1.2)	
Employment status	Unable to work due to ill health	20 (27.7)		
	Full-time employment	16 (22.2)		
	Retired	15 (20.8)		
	Part-time employment	7 (9.7)		
	Not working (other)	7 (9.7)		
	Self-employed	4 (5.55)		
Education	School	25 (34.7)		
	College	17 (23.6)		
	University	15 (20.8)		
	Postgraduate qualification	9 (12.5)		

### Hospital anxiety and depression scale (HADS)

**Fig. 1:**
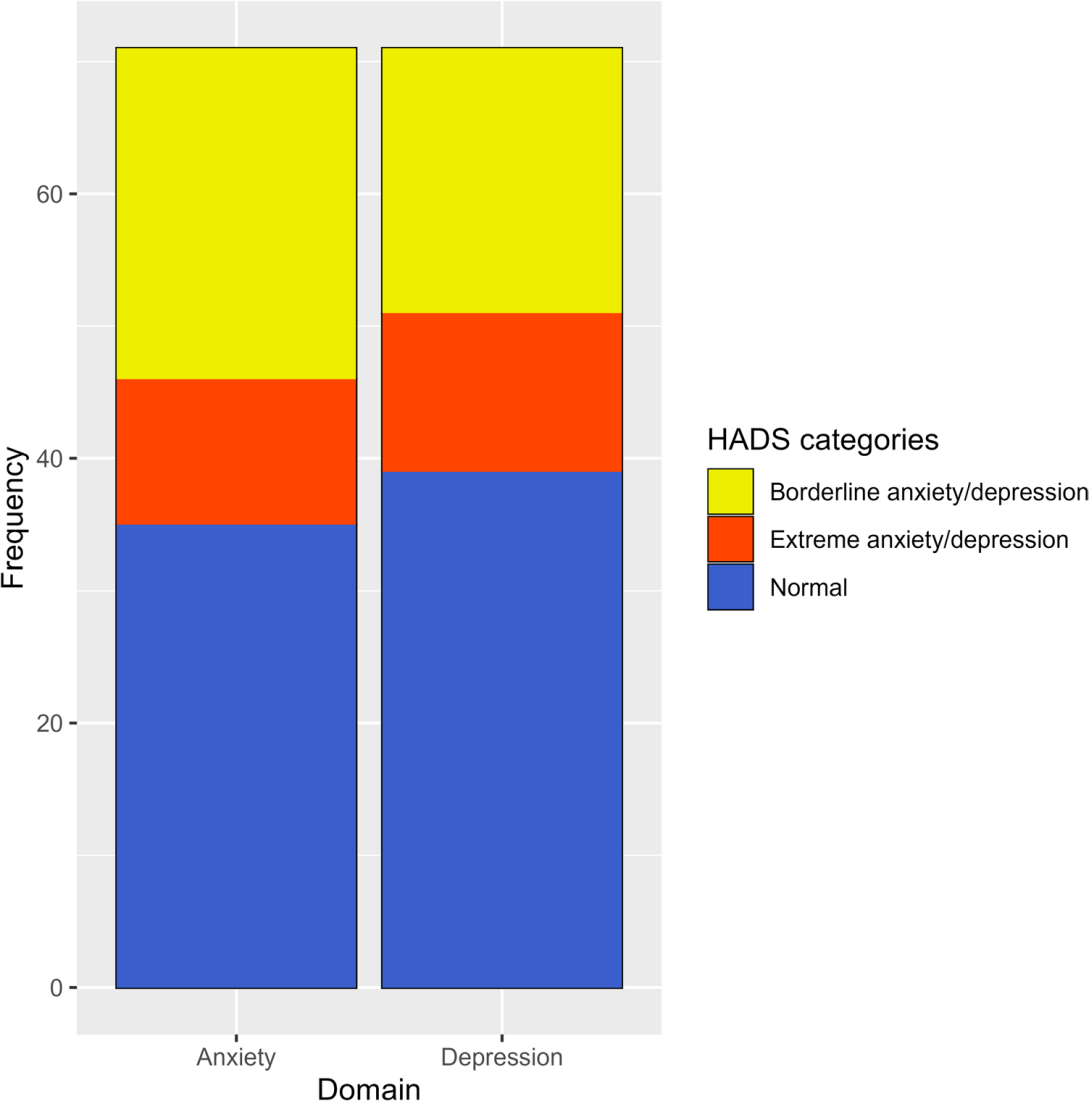
Stacked bar chart representing HADS categories

The median cumulative anxiety score for participants was 8.0 [IQR 4.0–14.0). Thirty-five participants (49.3%) were within the normal range, with 11 (15.5%) being categorised with borderline anxiety and 25 (35.2%) being categorised as extreme anxiety. The median cumulative depression score for participants was 7.0 (IQR 3.0–11.0). Thirty-nine (54.9%) participants were within the normal range, 12 (16.9%) were categorised with borderline depression and 20 (29.7%) were categorised with extreme depression. The HADS scores for the cohort are represented in the form of a stacked bar chart in Fig. 1. Additionally, Table S1, in the supplementary material, provides a breakdown of responses from the HADS questionnaire.

The median cumulative anxiety score for participants undergoing a craniectomy for TBI was 7.5 (range 1 – 19), tumour was 5.0 (range 0 – 14), and infected bone flap was 8.0 (range 3 – 17). There was no significant difference across median cumulative anxiety scores when stratified by craniectomy indication (*p* = *0.712).*

The median cumulative depression score for participants undergoing a cranioplasty for TBI was 8.0 (range 0 – 15), tumour was 4.0 (range 0 – 15), and infected bone flap was 10.0 (3 – 18). There was no significant difference across median cumulative depression score when stratified by craniectomy indication (*p* = *0.357).*

Table S3, in the supplementary material, provides a summary of results from the HADS questionnaire stratified by craniectomy indication.

### EuroQoL-5D-5L (EQ-5D)

Twenty-nine participants (41.4%) reported no mobility issues, 14 (20.0%) reported slight and 14 (20.0%) reported severe mobility issues. Forty-five (64.3%) participants reported no problems in washing or dressing themselves, with ten (14.3%) participants reporting slight and 6 (8.6%) reporting severe problems in washing or dressing themselves. Twenty-two (31.4%) participants had no problems doing their usual activities, however, 18 (25.7%) patients reported slight and 12 (17.1%) reported severe disruption in carrying out their usual activities. Twenty (29.0%) participants reported no pain or discomfort, however, 24 (34.8%) reported slight and 21 (30.4%) reported moderate pain or discomfort. The median VASc for the cohort was 70.0 [IQR 43.0–83.50). Summary data for the EQ-5D responses is shown in Table 2.

**Table 2:** Breakdown of EQ-5D domains

EQ – 5D	Score
Mobility:	
- I have no problems in walking about	− 29 (41.4)
- I have slight problems in walking about	− 14 (20.0)
- I have moderate problems in walking about	− 10 (14.3)
- I have severe problems in walking about	− 14 (20.0)
- I am unable to walk about	− 3 (4.3)
Self-care:	
- I have no problems washing or dressing myself	− 45 (64.3)
- I have slight problems washing or dressing myself	− 10 (14.3)
- I have moderate problems washing or dressing myself	− 5 (7.1)
- I have severe problems washing or dressing myself	− 6 (8.6)
- I am unable to wash or dress myself	− 4 (5.7)
Usual activities	
- I have no problems doing my usual activities	− 22 (31.4)
- I have slight problems doing my usual activities	− 18 (25.7)
- I have moderate problems doing my usual activities	− 9 (12.9)
- I have severe problems doing my usual activities	− 12 (17.1)
- I am unable to do my usual activities	− 9 (12.9)
Pain/discomfort:	
- I have no pain or discomfort	− 20 (29.0)
- I have slight pain or discomfort	− 24 (34.8)
- I have moderate pain or discomfort	− 21 (30.4)
- I have severe pain or discomfort	− 2 (2.9)
- I have extreme pain or discomfort	− 2 (2.0)
Anxiety/depression:	
- I am not anxious or depressed	− 23 (33.3)
- I am slightly anxious or depressed	− 24 (34.8)
- I am moderately anxious or depressed	− 12 (17.4)
- I am severely anxious or depressed	− 9 (13.0)
- I am extremely anxious or depressed	− 1(1.4)
Visual analog scale (IQR)	70.00 (43.00 – 83.50)

The median VASc for participants undergoing cranioplasty for TBI was 72.5 (range 15 – 100), tumour was 80.0 (range 25 – 95) and infected bone flap was 70.0 (range 5 – 90). There was no significant difference in median VASc score (*p* = *0.490)* across craniectomy indications. Patients undergoing craniectomy for cerebral infarct or other vascular pathology had significantly more impaired self-care (Cerebral infarct median 4.0, vascular pathology median 3.0) and daily activity (cerebral infarct median 4.5, vascular pathology median 4.5) domains. The remaining median scores across the EQ-5D HRQoL domains had no significant difference when comparing craniectomy indication. Table S3*,* in the supplementary material, provides a summary of EQ-5D domains stratified by craniectomy indication.

### RAND short form-36 questionnaire (SF-36)

The three highest scoring (better) HRQoL domains were physical functioning (Median 70.0, IQR 25.0–90.0), emotional well-being (Median 68.0, IQR 44.0 – 84.0), and pain (Median 67.5, IQR 45.0 – 90.0). The three lowest (worst) performing domains were general health (Median 45.0, IQR 25.0 – 70.0), energy/fatigue (Median 40.0, IQR 25.0 – 65.0), and role limitations for physical health (Median 25.0, Range 0.0 – 100.0). Table 3 provides summary data for all SF-36 domains. There was no significant difference in median score across the SF-36 HRQoL domains when comparing craniectomy indications. Table S3*,* in the supplementary material, provides a summary of SF-36 domains stratified by craniectomy indication.

**Table 3:** Breakdown of individual SF-36 domains

Domain	Median (IQR)
Physical functioning	70.0 (25.0 – 90.0)
Role limitations due to physical health	25.0 (0.0 – 100.0)
Role limitations due to emotional problems	66.7 (66.7 – 100.0)
Energy/Fatigue	40.0 (25.0 – 65.0)
Emotional well-being	68.0 (44.0 – 84.0)
Social functioning	62.5 (25.0 – 87.5)
Pain	67.5 (45.0 – 90.0)
General health	45.0 (25.0 – 70.0)

### Derriford appearance scale (DAS-24)

The median (IQR) total score for participants was 42.0 (32.0–56.0). After categorizing the participants in four groups based on their total questionnaire score, 22 (31.0%) participants reported no or nearly no negative feelings towards their appearance, 27 (38.0%) reported feeling conscious sometimes or having slight negative feelings, 17 (23.9%) reported feeling conscious often and having a fair number of negative feelings and 5 (7.0%) reported feeling conscious almost always and having extremely negative feelings towards their appearance.

There was no significant difference across DAS-24 domains when comparing craniectomy indications. TableS3, in the supplementary material, provides a summary of DAS-24 results stratified by craniectomy indication.

## Discussion

This cross-sectional study describes the health-related quality of life following cranioplasty in a large cohort of patients using a battery of questionnaires. In our cohort, nearly 1 in 3 participants reported feeling extremely anxious or depressed, 1 in 6 reported feeling borderline anxious or depressed and 6 in 10 participants had a problem with mobility, self-care, pain or discomfort. Nearly 6 in 10 patients reported being conscious of their appearance or having negative feelings towards it.

Although cosmesis and appearance following cranioplasty has been explored by previous studies, all of them employed a study specific questionnaire. Appearance is expected to affect health-related quality of life especially in patients undergoing cranioplasty as the operation itself aims to repair a defect in the skull following a craniectomy. Previous studies that explored patient reported cosmetic results following cranioplasty reported satisfaction in appearance following cranioplasty in 60% to 90% of their cohort [12, 13]. The reported improvement is relative to baseline cosmetic appearance of a craniectomy defect and therefore precludes the impact of the cranioplasty on an individuals’ perception of appearance and how it may affect their social activities.

Results from the DAS-24 questionnaire demonstrated that the worst performing items were participants feeling irritable at home, adopting concealing gestures, distressed in supermarkets/department stores, feeling rejected, avoiding leaving the house, being in physical pain/discomfort and their appearance affecting their sex life. When these scores were categorised nearly 7 in 10 participants reported feeling conscious of their appearance at a given time-point. Poor scores in the above-mentioned domains would be expected to influence other aspects of participants’ lives including anxiety, depression, and general health. This is reflected by responses in the HADS depression domain where nearly one in three participants reported not looking forward to enjoying activities, losing interest in their appearance, feeling slowed down, not feeling cheerful, and not enjoying the activities they used to enjoy as much before. Additionally, results from SF-36 indicate impaired scores in domains of social functioning, emotional well-being, and role limitations due to emotional problems, all of which may be attributed to results from DAS-24 which revealed over 7 in 10 participants feeling conscious about their appearance. In addition, baseline reduction social functioning and emotional well-being would also be influenced by participants’ underlying pathology, of brain tumour, traumatic brain injury, and stroke.

Two of the five questionnaires administered directly explored anxiety or depression. HADS only consisted of two domains, anxiety, and depression, and explored these using multiple items. The median anxiety score is comparable to the median normative population scores from a UK cohort; however, the median depression score was twice the normative population value in this cohort [14]. Reasons for this increase in the depression score may be due to the underlying pathology, which includes TBI and underlying tumour which are known to have a negative impact on individuals’ mood [15–17]. Results from the DAS-24 revealed 7 in 10 participants were conscious of their physical appearance, which would be expected to have a negative impact on their emotional well-being. Additionally, participants responded during or right after the second COVID-19 lockdown was in place in the United Kingdom. Fancourt et al. described depression and anxiety symptoms in a cohort of 70,000 adults in the United Kingdom using the Generalised Anxiety Disorder Questionnaire (GAD-7) and Patient Health Questionnaire-9 (PHQ-9) and found the highest levels of depression and anxiety in the first COVID-19 lockdown however noticed a significant decline in scores across the second lockdown [18]. Longitudinal HRQoL results one-year post COVID-19 lockdown as described by Joensen et al. indicate that participants improved from their baseline scores however continue to have an impairment in HRQoL [19]. Results from these large-scale prospective studies indicate that the COVID-19 lockdown remained a confounding factor for our study when describing symptoms of anxiety and depression.

We found no difference in HRQoL as measured by HADS, SF-36, or DAS-24 when comparing indications for craniectomy in our cohort. Reasons for this may include a small sample size in each craniectomy indication cohort which may not achieve statistically significant differences, additionally, craniectomy indications represented in our cohort included TBI, tumour, infected bone flap, cerebral infarct, vascular, cystic lesions, or primary intracranial infection, all of which represent a high degree of morbidity for patients leading to a global decline in HRQoL, which is represented in the results of the overall cohort. Results from EQ-5D showed worse scores in domains of self-care and daily activity for patients who underwent a craniectomy due to cerebral infarct or other vascular pathology. Giese et al. previously compared HRQoL using EQ-5D across craniectomy indications and reported no significant difference between patients undergoing craniectomy for TBI. Infection, or intra-cerebral/subarachnoid haemorrhage, however found a worsened overall HRQoL for patients undergoing a craniectomy related to cerebral infarction/ischaemia. The results of our cohort are consistent with previously described HRQoL studies [20].

Previously, three studies employed the SF-36 to evaluate HRQoL following cranioplasty. One of these studies did not provide a breakdown of the domains and instead only provided a comparative overview comparing two materials [21]. Another study categorised their cohort into good and poor without providing scores for each domain, thus making comparisons to the normative population and other cranioplasty cohorts impossible [20]. The median score for the general health domain in our cohort was 50.0 which was significantly less when compared to a previous study, which reported a median general health score of 83.0 [22]. This large disparity could be attributed to the clinical profile of the participants: the study conducted by Worm et al. consisted of patients predominantly affected by TBI and among the 62 in the cohort, only 8 participants had other pathology. This contrasts with the Cranio-PRO cohort in which nearly one third of the cohort is affected by an underlying brain tumour which would lead to a decline in overall general health. The cohort mean VASc score was 70 which is similar to previous VASc scores of 63 and 60 reported in the literature [20, 23].

### Study limitations

There are several limitations to the study. Although the focus of the study was to explore HRQoL following cranioplasty and this was conveyed via the PIS it is not possible to delineate which aspects of HRQoL are being affected by the cranioplasty itself, or by participants’ underlying pathology. Accordingly, we distributed a range of PROMs evaluating HRQoL, health status, anxiety and depression and self-reported problems with appearance. This was a cross-sectional study which precludes casual inference or temporal evaluation of HRQoL changes following cranioplasty, and as such longitudinal assessment of these patients is required. To address this gap, we will plan a longitudinal prospective study administering PROMs pre-operatively (baseline) and at post-operative clinic follow-up appointments. As this was a cross-sectional study with the aim of summarising multiple HRQoL domains using descriptive statistics for patients following a cranioplasty, sensitivity analyses were not performed. As the response rate was 30% no statistical tests were performed to account for missing data to avoid overstating descriptive results. Additionally, the first set of questionnaires were sent out during the second COVID-19 lockdown in the UK and hence some of the responses, related to overall emotional well-being and social activities may have been impacted by the pandemic as discussed above. The overall participation rate of our cohort was 30%. Reasons for a low response rate could include the clinical profile of the patients undergoing cranioplasty, with most patients having an existing pathology such as a brain tumour or traumatic brain injury, which could lead to impaired performance status and impaired cognitive ability [24]. Finally, several patients contacted resided in care homes which further expands on their functional status, with these patients not having capacity to consent. This might have led to our results not including those with most severe pathology.

### Implications for clinical practice

As demonstrated by previous work, the cranioplasty operation has a major impact on patients’ long-term health-related quality of life. Clinicians could employ standardised PROM tools to better understand the patient perspective following the operation and explore factors that lead to an improvement or decline in HRQoL. Longitudinal HRQoL assessment of for patients starting prior to the cranioplasty can allow clinicians to identify deterioration in HRQoL and subsequently signpost patients to relevant well-being services.

## Conclusion

Cranioplasty is a major neurosurgical operation. Commonly affected HRQoL areas include overall health, pain and discomfort and the appearance of the cranioplasty itself. Standardised PROM tools should be employed to track patient perception of the operation and identify any deterioration in HRQoL as a result from it.

## Supplementary information

Below is the link to the electronic supplementary material.

ESM 1 (DOCX 28.0 KB)

## Data Availability

Breakdown of questionnaire data is provided in the study tables and supplementary file. Anonymised data-breakdown is available upon request.

## References

[1] Bjornson A et al (2019) A case series of early and late cranioplasty—comparison of surgical outcomes. Acta Neurochir (Wien) 161(3):467–472. 10.1007/s00701-019-03820-9 30715606 10.1007/s00701-019-03820-9 PMC6407742

[2] Shah AM, Jung H, Skirboll S (2014) Materials used in cranioplasty: a history and analysis. Neurosurg Focus 36(4):E19. 10.3171/2014.2.FOCUS13561 24684331 10.3171/2014.2.FOCUS13561

[3] Henry J, Taylor J, Amoo M, O’Brien DP (2021) Complications of cranioplasty in relation to material: systematic review, network meta-analysis and meta-regression. Neurosurgery 89(3):383–394. 10.1093/neuros/nyab180 34100535 10.1093/neuros/nyab180

[4] Datti R, Cavagnaro G, Camici S (1985) Stainless steel wire mesh cranioplasty: ten years’ experience with 183 patients (100 followed up). Acta Neurochir (Wien) 78(3–4):133–135. 10.1007/BF01808692 4091052 10.1007/BF01808692

[5] Millward CP et al. (2022) Cranioplasty with hydroxyapatite or acrylic is associated with a reduced risk of all-cause and infection-associated explantation. Br J Neurosurg.1–9.10.1080/02688697.2022.2077311 10.1080/02688697.2022.2077311 35608052

[6] Andrabi SM, Sarmast AH, Kirmani AR, Bhat AR (2017) Cranioplasty: Indications, procedures, and outcome - an institutional experience. Surg Neurol Int 8:91. 10.4103/sni.sni_45_17 10.4103/sni.sni_45_17 PMC5461575 28607825

[7] Mustafa MA et al. (2023) Health-related quality of life following cranioplasty – a systematic review. Br J Neurosurg. 1–11. 10.1080/02688697.2023.2202244. 10.1080/02688697.2023.2202244 37265087

[8] Hays RD, Sherbourne CD, Mazel RM (1993) The RAND 36-item health survey 1.0. Health Econ 2(3):217–227. 10.1002/hec.4730020305 8275167 10.1002/hec.4730020305

[9] Herdman M et al (2011) Development and preliminary testing of the new five-level version of EQ-5D (EQ-5D-5L). Qual Life Res 20(10):1727–1736. 10.1007/s11136-011-9903-x 21479777 10.1007/s11136-011-9903-x PMC3220807

[10] Snaith RP (2003) The hospital anxiety and depression scale. Health Qual Life Outcomes 1:29. 10.1186/1477-7525-1-29 12914662 10.1186/1477-7525-1-29 PMC183845

[11] Carr T, Moss T, Harris D (2005) The DAS24: a short form of the derriford appearance scale DAS59 to measure individual responses to living with problems of appearance. Br J Health Psychol 10(Pt 2):285–298. 10.1348/135910705X27613 15969855 10.1348/135910705X27613

[12] Honeybul S, Morrison DA, Ho KM, Lind CRP, Geelhoed E (2017) A randomized controlled trial comparing autologous cranioplasty with custom-made titanium cranioplasty. J Neurosurg 126(1):81–90. 10.3171/2015.12.JNS152004 26991387 10.3171/2015.12.JNS152004

[13] Giese H, Meyer J, Unterberg A, Beynon C, Engel M (2020) Polymethylmethacrylate patient-matched implants (PMMA-PMI) for complex and revision cranioplasty: analysis of long-term complication rates and patient outcomes. Brain Inj 34(2):269–275. 10.1080/02699052.2019.1683895 31657239 10.1080/02699052.2019.1683895

[14] Breeman S, Cotton S, Fielding S, Jones GT (2015) Normative data for the hospital anxiety and depression scale. Qual Life Res 24(2):391–398. 10.1007/s11136-014-0763-z 25064207 10.1007/s11136-014-0763-z

[15] Keshwara SM et al (2023) Quality of life outcomes in incidental and operated meningiomas (QUALMS): a cross-sectional cohort study. J Neurooncol 161(2):317–327. 10.1007/s11060-022-04198-y 36525165 10.1007/s11060-022-04198-y PMC9756745

[16] Huang J et al (2017) Association between depression and brain tumor: a systematic review and meta-analysis. Oncotarget 8(55):94932–94943. 10.18632/oncotarget.19843 29212279 10.18632/oncotarget.19843 PMC5706925

[17] Juengst SB, Kumar RG, Wagner AK (2017) A narrative literature review of depression following traumatic brain injury: prevalence, impact, and management challenges. Psychol Res Behav Manag 10:175–186. 10.2147/PRBM.S113264 28652833 10.2147/PRBM.S113264 PMC5476717

[18] Fancourt D, Steptoe A, Bu F (2021) Trajectories of anxiety and depressive symptoms during enforced isolation due to COVID-19 in England: a longitudinal observational study. TLancet Psychiatry 8(2):141–149. 10.1016/S2215-0366(20)30482-X 33308420 10.1016/S2215-0366(20)30482-X PMC7820109

[19] Joensen A, Danielsen S, Andersen PK, Groot J, Strandberg-Larsen K (2022) The impact of the initial and second national COVID-19 lockdowns on mental health in young people with and without pre-existing depressive symptoms. J Psychiatr Res 149:233–242. 10.1016/j.jpsychires.2022.03.001 35290818 10.1016/j.jpsychires.2022.03.001 PMC8902858

[20] Giese H, Antritter J, Unterberg A, Beynon CAO-G ( 2021) Long-term results of neurological outcome, quality of life, and cosmetic outcome after cranioplastic surgery: a single center study of 202 patients. Front Neurol 12:702339. 10.3389/fneur.2021.702339 10.3389/fneur.2021.702339 PMC8329417 34354667

[21] Lindner D, Schlothofer-Schumann K, Kern B-C, Marx O, Müns A, Meixensberger J (2017) Cranioplasty using custom-made hydroxyapatite versus titanium: a randomized clinical trial. J Neurosurg 126(1):175–183. 10.3171/2015.10.JNS151245 26918471 10.3171/2015.10.JNS151245

[22] Worm PV, Finger G, Ludwig do Nascimento T, Rynkowski CB, and Collares MVM (2019) The impact of cranioplasty on the patients quality of life. J Cranio-Maxillofacial Surg 47(5):715–719. 10.1016/j.jcms.2019.01.040 10.1016/j.jcms.2019.01.040 30803855

[23] Henker C, Hoppmann M-C, Sherman MUS, Glass A, Piek J (2018) Validation of a novel clinical score: the rostock functional and cosmetic cranioplasty score. J Neurotrauma 35(8):1030–1036. 10.1089/neu.2017.5512 29256820 10.1089/neu.2017.5512

[24] Zucchella C, Bartolo M, Di Lorenzo C, Villani V, Pace A (2013) Cognitive impairment in primary brain tumors outpatients: a prospective cross-sectional survey. J Neurooncol 112(3):455–460. 10.1007/s11060-013-1076-8 23417320 10.1007/s11060-013-1076-8

