# Supplementary material for: Health-related quality of life following cranioplasty: a cross-sectional cohort study – Cranio-PRO
Source: Neurosurg Rev. 2025 Jan 14;48(1):45. doi: 10.1007/s10143-024-03158-6 (PMC11732772; doi:10.1007/s10143-024-03158-6)
Supplement: Supplementary file 1 — (DOCX 28.0 KB) [file 10143_2024_3158_MOESM1_ESM.docx]

**Supplementary material**

**Table S1: Breakdown of the hospital and anxiety depression questionnaire**

| **Domain** | **Number** |
| --- | --- |
| **Anxiety** |  |
| I feel tense or ‘wound up’:   - Not at all - Time to time, occasionally - A lot of the time - Most of the time | - 7 (15.6) - 20 (44.4) - 13 (28.9) - 5 (11.1) |
| I get a sort of frightened feeling like something awful is about to happen:   - Not at all - A little, but it doesn’t worry me - Yes, but not too badly - Very definitely and quite badly | - 13 (28.9) - 17 (37.8) - 9 (20.0) - 6 (13.3) |
| Worrying thoughts go through my mind:   - Only occasionally - From time to time but not too often - A lot of the time - A great deal of the time | - 10 (22.7) - 14 (31.8) - 9 (20.5) - 11 (25.0) |
| I can sit at ease and feel relaxed:   - Not at all - Not often - Usually - Definitely | - 2 (4.5) - 12 (27.3) - 14 (31.8) - 16 (36.4) |
| I get a sort of frightened feeling like ‘butterflies in the stomach’   - Not at all - Occasionally - Quite often - Very often | - 19 (43.2) - 16 (36.4) - 7 (15.9) - 2 (4.5) |
| I feel restless as if I have to be on the move:   - Not at all - Not every much - Quite a lot - Very much indeed | - 18 (40.9) - 10 (22.7) - 12 (27.3) - 4 (9.1) |
| I get sudden feelings of panic:   - Not at all - Not very often - Quite often - Very often indeed | - 10 (22.7) - 21 (47.7) - 10 (22.7) - 3 (6.8) |
| **Depression** |  |
| I still enjoy the things I used to enjoy:   - Not at all - Only a little - Not quite so much - Definitely as much | - 8 (17.8) - 9 (20.0) - 15 (33.3) - 13 (28.9) |
| I can laugh and see the funny side of things:   - Not at all - Definitely not so much now - Not quite so much now - As much as I always could | - - - 9 (20.0) - 13 (28.9) - 23 (51.1) |
| I feel cheerful:   - Not at all - Not often - Sometimes - Most of the time | - 1 (2.2) - 9 (20.0) - 19 (42.2) - 16 (35.6) |
| I feel as if I am slowed down:   - Not at all - Sometimes - Very often - Nearly all of the time | - 11 (24.4) - 10 (22.2) - 17 (37.8) - 7 (15.6) |
| I have lost interest in my appearance:   - I take just as much care as ever - I may not take quite as much care - I don’t take as much care as I should - Definitely | - 21 (48.8) - 10 (23.3) - 7 (16.3) - 5 (11.6) |
| I look forward with enjoyment to things:   - Hardly at all - Definitely less than I used to - Rather less than I used to - As much as I ever did | - 3 (6.8) - 14 (31.8) - 11 (25.0) - 16 (36.4) |
| I can enjoy a good book or radio or TV programme:   - Very seldom - Not often - Sometimes - Often | - 2 (4.5) - 6 (2.5) - 9 (3.8) - 27 (11.3) |
| **Cumulative score** | |
| **Anxiety**:   - Median (Range) - 0 – 7 (Normal) - 8 – 10 (Borderline) - 11 and above (Extreme) | - 6 (0 – 19) - 24 55.8) - 7 (16.3) - 12 (27.9) |
| **Depression**:   - Median - 0 – 7 (Normal) - 8 – 10 (Borderline) - 11 and above (Extreme) | - 7 (0 – 18) - 22 (51.2) - 8 (18.6) - 13 (30.2) |

**Table S2: Breakdown of the Derriford Appearance Scale questionnaire**

| **Item number** | **Item summary** | **Median (IQR)** |
| --- | --- | --- |
| a) | Feeling confident | 2.00 (1-3) |
| b) | Distress at reflection | 2.00 (1-3) |
| c) | Irritable at home | 1.00 (1-2) |
| d) | Feel hurt | 2.00 (1-3) |
| e) | Self-consciousness affects work | 1.50 (1-2) |
| f) | Distressed at beach | 2.00 (1-2) |
| g) | Misjudged due to appearance | 2.00 (1-3.3) |
| h) | Feel/feminine/masculine | 3.00 (2-4) |
| i) | Self-conscious of appearance | 2.00 (1-3) |
| j) | Feel irritable | 2.00 (1-3) |
| k) | Adopt concealing gestures | 1.00 (1-2) |
| l) | Avoid communal changing | 2.00 (1-4) |
| m) | Distressed in supermarkets/dept. stores | 1.00 (1-3) |
| n) | Feel rejected | 1.00 (1-2.8) |
| o) | Avoid undressing with partner | 1.00 (1-1.5) |
| p) | Distressed playing sport/games | 2.00 (1-4) |
| q) | Close into shell | 2.00 (1-2.8) |
| r) | Distressed at social events | 2.00 (1-4) |
| s) | Distressed at social events | 2.00 (1-3) |
| t) | Feel normal | 3.00 (2-4) |
| u) | Affects sex life | 1.00 (1-3) |
| v) | Avoid leaving house | 1.00 (1-2) |
| w) | Distressed at other remarks about appearance | 2.00 (1.8-3.3) |
| x) | Avoid pubs/restaurants | 1.00 (1-4) |
| y) | Physical pain/discomfort | 1.00 (1-2) |
| z) | Limiting physical ability to do things | 2.00 (1-3.0) |
|  | Median total score | 38.00 (31 – 54) |
|  | Score categorized into frequency of appearance problems   - Never/almost never - Sometimes/slightly - Often/A fair amount - Almost always/extremely | - 15 (34.1%) - 16 (36.4%) - 10 (22.7%) - 3 (6.8%) |

**Table S3: Breakdown of HRQoL domains as measured by HADS, SF-36, EQ-5D, and DAS-24 stratified by craniectomy** **indication**

| **Variable** | **Sub-variable** | ***P-Value* (95% CI)** |  |
| --- | --- | --- | --- |
| **HADS** | Cumulative anxiety* | TBI (7.5), Tumour (5.0), Cerebral infarct (17.0), Vascular (6.0), Cystic lesion (5.0), Infected bone flap (8.0), Primary intracranial infection (n/a), Other (7.0) | 0.712 |
| Cumulative depression* | TBI (8.0), Tumour (4.0), Cerebral infarct (16.0), Vascular (10.0), Cystic lesion (5.0), Infected bone flap (10.0), Primary intracranial infection (12.0), Other (2.0) | 0.102 |  |
| **SF-36** | Physical functioning | TBI (60.0), Tumour (77.5), Cerebral infarct (10.0), Vascular (40.0), Cystic lesion (50.0), Infected bone flap (80.0), Primary intracranial infection (20.0), Other (77.5) | 0.323 |
|  | Role limitation - physical health | TBI (25.4), Tumour (50.1), Cerebral infarct (1.0), Vascular (50.0), Cystic lesion (0.3), Infected bone flap (25.5), Primary intracranial infection (0.3), Other (50.0) | 0.523 |
|  | Role limitation - emotional health | TBI (33.3), Tumour (100.0), Cerebral infarct (0.0), Vascular (66.7), Cystic lesion (66.7), Infected bone flap (100.0), Primary intracranial infection (N/A), Other (100.0) | 0.328 |
|  | Energy/  Fatigue | TBI (42.5), Tumour (62.5), Cerebral infarct (40.0), Vascular (55.6), Cystic lesion (25.0), Infected bone flap (25.0), Primary intracranial infection (N/A), Other (70.0) | 0.222 |
|  | Emotional well-being | TBI (72.0), Tumour (76.0), Cerebral infarct (24.0), Vascular (58.0), Cystic lesion (60.0), Infected bone flap (44.0), Primary intracranial infection (88.0), Other (84.0) | 0.246 |
|  | Social functioning | TBI (56.3), Tumour (87.5), Cerebral infarct (12.5), Vascular (25.0), Cystic lesion (37.5), Infected bone flap (25.0), Primary intracranial infection (25.0), Other (62.5) | 0.436 |
|  | Pain | TBI (90.0), Tumour (67.5), Cerebral infarct (22.5), Vascular (73.8), Cystic lesion (45.0), Infected bone flap (77.5), Primary intracranial infection (45.0), Other (67.5) | 0.104 |
|  | General health | TBI (60.0), Tumour (55.0), Cerebral infarct (20.0), Vascular (15.0), Cystic lesion (30.0), Infected bone flap (45.0), Primary intracranial infection (15.0), Other (20.0) | 0.238 |
| **EQ-5D** | Mobility* | TBI (1.0), Tumour (2.0), Cerebral infarct (4.0), Vascular (3.5), Cystic lesion (2.0), Infected bone flap (2.0), Primary intracranial infection (4.0), Other (1.5) | 0.077 |
|  | Self-care* | TBI (1.0), Tumour (1.0), Cerebral infarct (4.0), Vascular (3.0), Cystic lesion (1.0), Infected bone flap (1.0), Primary intracranial infection (1.0), Other (1.0) | 0.006 (CI 0.030 - 0.363) |
|  | Usual activities* | TBI (2.0), Tumour (2.0), Cerebral infarct (4.5), Vascular (4.5), Cystic lesion (1.0), Infected bone flap (2.0), Primary intracranial infection (1.0), Other (1.0) | 0.007 (CI 0.026 - 0.357) |
|  | Anxiety/  Depression* | TBI (2.0), Tumour (2.0), Cerebral infarct (3.0), Vascular (2.0), Cystic lesion (1.0), Infected bone flap (2.0), Primary intracranial infection (2.0), Other (2.0) | 0.457 |
|  | Pain/  Discomfort* | TBI (2.0), Tumour (2.0), Cerebral infarct (3.0), Vascular (2.0), Cystic lesion (2.0), Infected bone flap (2.0), Primary intracranial infection (2.0), Other (3.0) | 0.281 |
|  | VAS | TBI (72.5), Tumour (80.0), Cerebral infarct (33.5), Vascular (45.0), Cystic lesion (65.0), Infected bone flap (70.0), Primary intracranial infection (60.0), Other (67.5) | 0.396 |
| **DAS-24** | Appearance score* | TBI (2.0), Tumour (2.0), Cerebral infarct (2.5), Vascular (3.0), Cystic lesion (3.0), Infected bone flap (2.0), Primary intracranial infection (3.0), Other (2.0) | 0.319 |

***Higher score** **indicates a more impaired HRQoL**
